# Studying the Structures of Relaxed and Fuzzy Interactions: The Diverse World of S100 Complexes

**DOI:** 10.3389/fmolb.2021.749052

**Published:** 2021-10-11

**Authors:** Péter Ecsédi, Gergő Gógl, László Nyitray

**Affiliations:** ^1^ Department of Biochemistry, Eötvös Loránd University, Budapest, Hungary; ^2^ Department of Integrative Structural Biology, Institut de Génétique et de Biologie Moléculaire et Cellulaire (IGBMC), INSERM U1258/CNRS UMR 7104/Université de Strasbourg, Illkirch, France

**Keywords:** S100 proteins, fuzzy interactions, X-ray crystallography, NMR, annexin A2, p53, RSK1, NM2A

## Abstract

S100 proteins are small, dimeric, Ca^2+^-binding proteins of considerable interest due to their associations with cancer and rheumatic and neurodegenerative diseases. They control the functions of numerous proteins by forming protein–protein complexes with them. Several of these complexes were found to display “fuzzy” properties. Examining these highly flexible interactions, however, is a difficult task, especially from a structural biology point of view. Here, we summarize the available *in vitro* techniques that can be deployed to obtain structural information about these dynamic complexes. We also review the current state of knowledge about the structures of S100 complexes, focusing on their often-asymmetric nature.

## Introduction

In nature, most protein domains adopt a well-defined tertiary structure and are considered “ordered.” The formation of such globular structures was used to be considered necessary for proteins to display functional activity—and thus came the term “structure–function relationship.” However, forming a globular structure is no longer regarded as a universal requirement for proteins, since several regions and even full proteins have been shown to be disordered and functional at the same time (Dunker et al., 2001; Gsponer and Babu, 2009; Tompa, 2012; van der Lee et al., 2014). These regions and proteins are usually referred to as intrinsically disordered regions (IDRs) and intrinsically disordered proteins (IDPs), respectively. IDRs are very often targets of post-translational modifications (PTMs) (Gsponer and Babu, 2009; van der Lee et al., 2014) and contain linear motifs that participate in many protein–protein interactions (PPIs) (Weatheritt et al., 2012). Note here that although they lack a fixed conformation, typically in the absence of their interaction partners, many IDPs are promiscuous binders, complexed *via* multiple binding scenarios (Weatheritt et al., 2012; Uversky, 2013). Certain regions of a given IDR interact with its binding partner, while other sections do not necessarily form interactions and remain flexible. Such a complex can thus be described as a heterogeneous ensemble of different conformations (Arbesú et al., 2018). These IDR complexes represent a continuum of fuzzy complexes from the rather rigid polymorphic complexes, in which the bindings of IDRs are fixed with only a few stable alternative conformations, to the highly dynamic random complexes, where numerous binding scenarios can appear at nearly equal energy levels (Tompa and Fuxreiter, 2008; Uversky and Dunker, 2010; Sharma et al., 2015; Arbesú et al., 2018; Miskei et al., 2020). These binding modes are more permissive than the lock-and-key-like, the induced-fit, or the fluctuation-induced interaction modes, typically used to picture enzyme–ligand interactions (Tripathi and Bankaitis, 2017). In this traditional picture, the binding of a ligand to an enzyme can occur only for one conformation; thus, the association is under sequential and conformational control, and the complex can be described with a well-defined structure (Savir and Tlusty, 2007; Nussinov et al., 2014). In contrast, IDRs and IDPs can—due to their high conformational flexibility—easily adapt to the interaction grooves of their binding partners (Gsponer and Babu, 2009).

**FIGURE 1:**
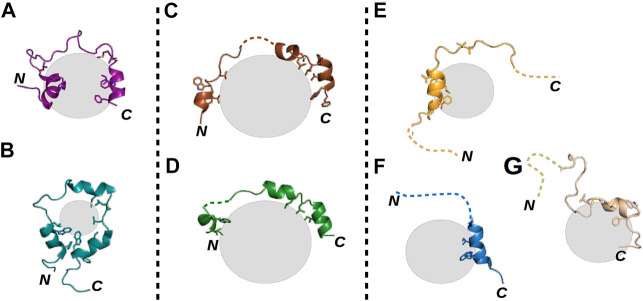
Structural illustrations of various fuzzy interactions made by the p53 TAD. Gray spheres represent the different partner proteins, the labels N and C mark the locations of the termini of each p53 TAD chain, and dashed lines represent highly flexible disordered regions of the TAD in the respective complexes. **(A)** TAZ–p53 TAD^1–56^ (PDB ID: 5HOU) (Krois et al., 2016) and **(B)** CRB–p53 TAD^13–61^ (PDB ID: 2L14) (Lee et al., 2010) complexes represent polymorphic interactions, where nearly the whole TAD is bound with few alternative conformations. **(C)** S100P–p53 TAD^17–56^ (PDB ID: 7NMI) and **(D)** S100A4–p53 TAD^17–56^ (PDB ID: 6T58) (Ecsédi et al., 2020) complexes show the characteristics of clamp-like fuzzy interactions where the TAD is anchored at several binding points, while the linking regions remain flexible and appear in numerous conformations. Finally, **(E)** p300–p53 TAD^1–39^ (PDB ID: 2K8F) (Feng et al., 2009), **(F)** TFIIH–p53 TAD^1–55^ (PDB ID: 2GS0) (Di Lello et al., 2006), and **(G)** HMG box–p53 TAD^1–93^ (PDB ID: 2LY4) (Rowell et al., 2012) complexes are good examples of flanking fuzzy interactions. Here, only a small portion of the TAD is binding the partner, while the sequences of the N- and C-termini remain disordered without any confinable structure.

A commonly studied example of an IDR is the N-terminal transactivation domain (TAD) of the tumor suppressor protein p53. Note that the only region of p53 having a stable tertiary structure is its DNA-binding domain (DBD); its other regions, including the TAD, remain disordered in the apo form (Joerger and Fersht, 2010). The TAD contains several PTMs and interaction sites (Gu and Zhu, 2012; Raj and Attardi, 2017) and is capable of forming polymorphic (Figures 1A,B), clamping (Figures 1C,D), and flanking (Figures 1E–G) fuzzy complexes with the same sequence (Di Lello et al., 2006; Feng et al., 2009; Lee et al., 2010; Rowell et al., 2012; Krois et al., 2016; Raj and Attardi, 2017; Ecsédi et al., 2020). The formation of these complexes is mostly driven by the two hydrophobic regions of the TAD (TAD1 and TAD2), while other residues outside these regions shape the specificity of the given interaction (Raj and Attardi, 2017; Dudás et al., 2020), being mainly anchored at a few positions when they participate in the binding and remaining flexible when not. Thus, one can suggest that the presence of fuzziness is a good indicator of the presence of multiple binding partners. The ability to form multiple interactions with the same part of a protein is highly advantageous. For example, such an ability allows p53, after its interaction with the MDM2 inhibitor is disrupted, to activate several signaling pathways simultaneously without having to have numerous domains or recognition motifs, each specific for only one partner. Moreover, MDM2-controlled inhibition in this case becomes more effective, as besides enhancing p53 degradation, MDM2 can operate as a competitive inhibitor (Moll and Petrenko, 2003).

In order to better understand the complex relationship between the structural features of biomolecules and their functions, both their ordered and disordered states have to be studied (Forman-Kay and Mittag, 2013). However, analyzing IDRs or fuzzy complexes is a difficult task, especially if structural information is sought, which requires the use of several different *in vitro* techniques. Over the years, we have studied PPIs of the S100 protein family, including the ones made by the p53 TAD, and obtained high-resolution structural data of several relaxed and fuzzy complexes. Here, we show how contemporary structural biology methods, together with other techniques deployed *in vitro*, can be used to study these flexible structures and also summarize our current knowledge about S100 complexes and their dynamics.

**FIGURE 2:**
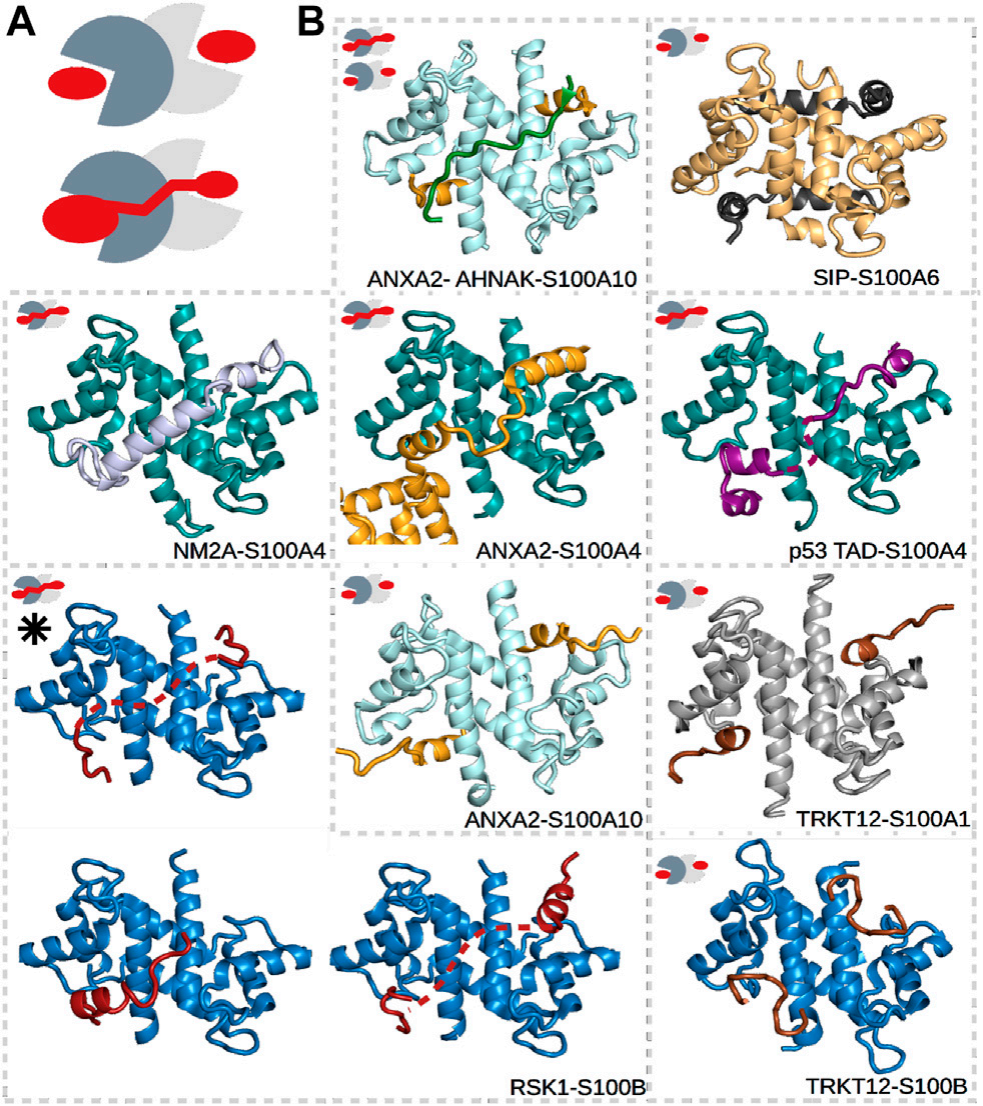
Different types of interactions in S100 complexes. **(A)** Schematic representations of symmetric **(top)** and asymmetric **(bottom)** interactions made by S100 proteins. The monomers of S100 proteins are represented in light and dark gray, while the partner is in red. **(B)** S100A1 (gray), S100A4 (deep teal), S100A6 (pale yellow), S100A10 (pale cyan), and S100B (sky blue) in complex with TRKT12 (brown, PDB: 1MQ1 and 2KBM) (McClintock and Shaw, 2003), RSK1 (red, PDB: 5CSN, 5CSJ, and 5CSI) (Gógl et al., 2016), NM2A (light blue, PDB: 3ZWH) (Kiss et al., 2012), ANXA2 (bright orange, PDB: 5LPU and 4HRE) (Oh et al., 2013; Ecsédi et al., 2017), SIP (black, PDB: 2JTT) (Lee et al., 2008), AHNAK (forest green, PDB: 4FTG) (Ozorowski et al., 2013), and p53 (deep purple, PDB: 6T58) (Ecsédi et al., 2020). Small pictograms show the nature of each interaction, i.e., whether it is symmetric or asymmetric. The black asterisk marks the structure of the RSK1–S100B complex determined in part using SAXS data. Dashed lines represent the expected locations of flexible regions, which were not visible during model building.

S100 proteins belong to the EF-hand Ca^2+^-binding superfamily (Kawasaki and Kretsinger, 2017; Heizmann, 1988), which appeared in early vertebrates and include at least 20 core members in humans. The genes of 16 of these members, namely, S100A1–A16, are closely clustered together in the same chromosome, while the genes of S100B, S100G, S100P, and S100Z are at different locations (Marenholz et al., 2004). The physiological functions of S100’s are still not well understood, but intracellularly, they are known to participate in processes such as proliferation, differentiation, apoptosis, Ca^2+^ homeostasis, and energy metabolism, while extracellular S100 proteins can activate surface receptors, G-protein–coupled receptors, or scavenger receptors (Marenholz et al., 2004; Donato et al., 2013). Their concentrations are detectably perturbed in the milieu affected by cancer growth, metastasis, angiogenesis (Bresnick et al., 2015), and rheumatic (Austermann et al., 2018) and neurodegenerative diseases (Marenholz et al., 2004; Heizmann et al., 2002; Cristóvão and Gomes, 2019). S100 proteins do not have enzymatic activity but instead control the functions of numerous proteins *via* PPIs. They have many established and potential interaction partners that can bind with low or high affinities, and not exclusively to one member of the family (Simon et al., 2020). This observed high promiscuity is likely due to the high sequential and structural similarities within the family (Heizmann et al., 2002), and due to that S100 proteins usually interact with the IDRs of their partners. Such partner proteins of S100’s, for which structures of the complexes have been determined, include the already mentioned p53 (Ecsédi et al., 2020; Fernandez-Fernandez et al., 2008; van Dieck et al., 2009a; Salama et al., 2008), motor protein non-muscle myosin 2A (NM2A) (Ecsédi et al., 2018; Kiss et al., 2016), Ca^2+^- and membrane-binding annexin A2 (ANXA2) (Liu et al., 2015; Ecsédi et al., 2017), ribosomal S6 kinase 1 (RSK1) (Gógl et al., 2016), the calcyclin-binding protein (SIP) (Lee et al., 2008), and the synthetic TRKT12 peptide (McClintock and Shaw, 2003) (Figure 2). Note that IDRs of other proteins, including centrosomal proteins such as FOP (Simon et al., 2020) and FOR20 (Sakane et al., 2017), have also been shown to interact with S100 proteins, but no structural data of these complexes are available yet.

### Binding Stoichiometry as an Indicator of Fuzziness

Dimeric S100’s are bivalent proteins, meaning that they have two specialized binding regions/pockets that are either identical or slightly different in the case of homo- and heterodimers, respectively (Heizmann et al., 2002). These binding surfaces function as anchor points for their partners but are buried in the absence of Ca^2+^. They become accessible only after Ca^2+^ binds to the EF-hands of the S100 proteins and induces a conformational rearrangement in them (Santamaria-Kisiel et al., 2006). Between the binding pockets, a shallow groove can be found at the dimeric interface, which is less specific with regard to the binding partners that it binds. Depending on the number of partners binding to a dimeric S100, symmetric or asymmetric interactions can occur (Simon et al., 2020). In symmetric complexes, two identical binding peptides occupy the Ca^2+^-dependent sites (e.g., ANXA2–S100A10 and TRKT12–S100B as shown in Figure 2) (Lee et al., 2008; McClintock and Shaw, 2003; Réty et al., 1999; Wright et al., 2008; Penumutchu et al., 2014; Gupta et al., 2013; Ozorowski et al., 2013; Rustandi et al., 2000; Oh et al., 2013). In asymmetric complexes, on the contrary, a single binding sequence utilizes both sites (e.g., ANXA2–S100A4, NM2A–S100A4, and RSK1–S100B as shown in Figure 2) (Kiss et al., 2012; Duelli et al., 2014; Gógl et al., 2016; Ecsédi et al., 2017; Ecsédi et al., 2020). In addition, the asymmetrically binding peptides can also use the intervening cleft between the binding pockets to various degrees. Some partners fully occupy it (e.g., NM2A–S100A4 in Figure 2), while others barely use it (e.g., RSK1–S100B in Figure 2). Thus, we have found that asymmetric S100 interactions are prone to a high degree of fuzziness.

A different kind of fuzziness may also appear in symmetric complexes. Although their binding stoichiometry has been shown *in vitro* to be 2:2, it may often be 2:1 (monomers of S100:number of partner molecules, respectively) in cells, where the concentrations of biomolecules are typically low (Marsh et al., 2015). In this case, the complex becomes asymmetric from a stoichiometric point of view because only a single interaction site becomes occupied in the bound conformation. Since biomolecular complex formation is often achieved through a two-step reaction, where first an electrostatic-driven “non-specific” encounter complex appears and later isomerizes into the bound conformations(s) (Schreiber and Fersht, 1996), it is possible that, in the case of such a stoichiometrically asymmetric S100 complex, a hopping mechanism could in principle be used to nevertheless produce an on-average structurally symmetric S100 complex. In such a case, the bound partner partially dissociates, but the resulting electrostatic encounter complex remains and re-isomerizes into a bound conformation again, where this time the partner utilizes the opposite binding pocket of the S100 dimer. This way, a dynamic exchange could occur between the interaction partner and the S100 dimer in the bound complex.

The binding modes of S100 proteins are reminiscent of the interaction properties of 14-3-3 proteins, which can also form homo- or heterodimers and have a specific binding pocket in each monomer that can accommodate various sequences (Fu et al., 2000). Members of this protein family recognize phosphorylated serine and threonine residues and are part of a regulatory network together with protein kinases and phosphatases that modify the binding partners of 14-3-3 proteins (Johnson et al., 2010; Pennington et al., 2018; Gogl et al., 2021; Munier et al., 2021). Similarly to the case of S100 proteins, both symmetric and asymmetric 14-3-3 interactions have been described (Molzan and Ottmann, 2012; Stevers et al., 2016; Sluchanko et al., 2017; Guillory et al., 2020; Kalabova et al., 2020; Munier et al., 2021). In the case of symmetric binding, two identical phosphorylated peptides occupy both binding surfaces of the 14-3-3 protein dimer, while in asymmetric interactions, a single sequence utilizes both binding pockets, doubling the number of residues involved and strengthening the interaction (Stevers et al., 2016). In the latter case, the intermediate sections between the binding grooves do not participate in the interaction, and consequently, the linker between the two anchored sections of the doubly phosphorylated partner remains flexible. Thus, one can easily assume that, in the case of 14-3-3 proteins, asymmetry and fuzziness can be an indicator of relatively strong interactions. Note that the length of the partner peptide or IDR can also influence the stoichiometry of the binding in both the S100 and 14-3-3 families, since an adequate number of residues are needed to connect the two bound sections.

Asymmetry and fuzziness can be observed in other types of bivalent systems, such as in the case of the tandem SH2 domains of SH2-containing protein tyrosine phosphatase-2 (SHP2). Here, the apparent binding affinity of a disordered partner peptide with tandem binding motifs shows increased affinity when an optimal linker connects the motifs (Eck et al., 1996). In this case, the two SH2 domains, although they are not part of dimeric interactions but part of the same molecule, capture two cognate parts of a larger IDR where the relative orientations of both the domains and the binding ligands vary considerably. While, according to the above-described examples, asymmetry can be an indicator of fuzziness, it is not necessarily so in every system. AKAP motifs, for instance, bind to PKA R-subunit dimers asymmetrically, and yet with no apparent fuzzy properties (Newlon et al., 2001; Gold et al., 2006). However, it is also important to note here that, in PKA, the protomers (monomeric units) do not form the binding pockets alone, as the sole binding pocket is located at the dimerization interface.

For the above reasons, analyzing the stoichiometry of PPIs where an IDR is captured on more than one distinct binding site is highly recommended. The stoichiometry of molecular interactions can be measured using various biochemical interaction assays, such as isothermal titration calorimetry (ITC) (Matsuyama et al., 2017; Simon et al., 2020), surface plasmon resonance (SPR) (Day et al., 2013), NMR spectroscopy (Ziarek et al., 2011), and fluorescence polarization (FP) (Dou et al., 2004). Each technique has its own advantages and drawbacks, but ITC is most widely used because, here, stoichiometry can be more precisely determined at high concentrations, and because the instrumentation for this technique is easier to operate than is that for NMR spectroscopy. Besides stoichiometry, ITC can be used to determine the values of thermodynamic parameters of interactions by analyzing the temperature changes caused by the formation of the complex. These results could also provide some information about the flexibility of the studied interaction. Such information can be provided because in the case of an IDP, an entropic cost for its engagement in a binding interaction can be expected, but at the same time, such a cost can be compensated by either optimizing the enthalpy gain (ΔH° < 0) or minimizing this entropy loss (–TΔS° ≈ 0) (Arbesú et al., 2018). Since fuzziness is a consequence of an IDP minimizing its energetically unfavorable folding (the more disordered it remains, the lesser the entropy it loses) (Hadži et al., 2017), one can assume that the greater the enthalpy gain is, the less relaxed the interaction can be, since there is no need to minimize the entropy loss caused by folding.

ITC was used to determine the stoichiometry of the interaction between NM2A and S100A4 (Kiss et al., 2012; Badyal et al., 2011); this interaction, besides other S100 interactions with p53 (van Dieck et al., 2009a), MDM2 (van Dieck et al., 2010), and a tripartite S100A10–ANXA2–AHNAK assembly (Ozorowski et al., 2013), was the first shown to be asymmetric. Previous high-resolution structures of S100 complexes were exclusively found to be symmetric (Lee et al., 2008; Réty et al., 1999; Rustandi et al., 2000). The first atomic-resolution structure determined for an asymmetric ternary complex in the S100 family was that of the previously mentioned ANXA2–S100A10–AHNAK complex (2:2:1) (Ozorowski et al., 2013) (Figure 2), while the first one with 1:2 stoichiometry was that of the NM2A–S100A4 complex, and it was obtained in parallel studies using both X-ray crystallography (Kiss et al., 2012) (Figure 2) and NMR spectroscopy (Elliott et al., 2012). The earlier observations, which showed only symmetric complexes, were of complexes including for the most part much shorter peptide ligands for structural analysis (∼20 residues), while in the case of the later observations of asymmetric complexes, longer peptides were used (≥40 residues)—this difference highlights the importance of a well-determined minimal binding sequence because, as previously described, the length of the partner sequence can affect the stoichiometry. Several additional crystal structures of asymmetric complexes have since been determined by our group (Gógl et al., 2016; Ecsédi et al., 2017; Dudás et al., 2020; Ecsédi et al., 2020). We have found that even in the case of the S100A4–NM2A complex, which involves one of the strongest asymmetric S100 interactions, with a sub-nanomolar dissociation constant (Ecsédi et al., 2018), minor differences between its different high-resolution structures occur (Elliott et al., 2012; Kiss et al., 2012; Duelli et al., 2014). The C-terminal tail of the NM2A peptide (1923–1935) remains unstructured in the complex, unlike its N-terminus. The interaction between NM2A and S100A4 is driven by the hydrophobic residues of the C-terminal region (L1926, F1928, V1927, and V1928), but without a constant binding pattern: some structures showed L1926 and V1928 oriented, respectively, into and out of the binding pocket of S100A4, while other structures showed the opposite. Thus, the C-terminal region can appear at least in two bound conformations.

**FIGURE 3:**
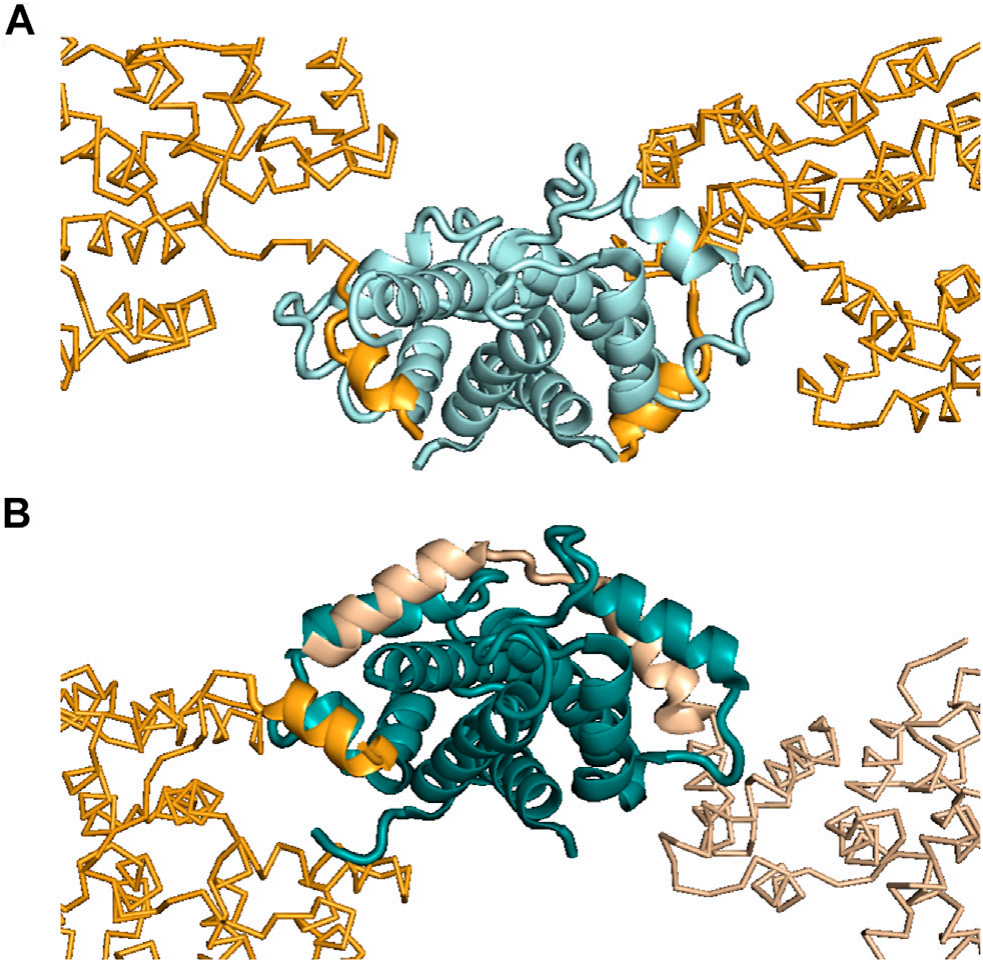
Interactions made between ANXA2 and different S100 proteins. **(A)** Structure showing ANXA2–S100A10 interactions (PDB: 4HRE) (Oh et al., 2013). **(B)** Structure showing ANXA2–S100A4 interactions (PDB: 5LPU) (Ecsédi et al., 2017). S100A10 is presented in pale cyan, while S100A4 is shown in deep teal. The interacting N-terminus of ANXA2 is shown in bright orange (S100A10-type, symmetric binding) and in wheat (S100A4-type, asymmetric binding). Inspection of the crystal structures showed two ANXA2 molecules bound to S100A10 symmetrically (bright orange) and two bound to S100A4 asymmetrically (wheat and bright orange). However, in the case of S100A4, ITC studies showed the interaction to be formed with only one ANXA2 molecule in solution, i.e., not with two ANXA2 molecules. This arrangement would appear to require this one molecule to occupy both binding pockets of the S100A4 dimer, as was observed in the crystal structure [see wheat-colored ANXA2 on the right side of panel **(B)**]. Based on the ITC results, the binding of the other ANXA2 molecule [bright orange, similar to the S100A10–ANXA2 interaction, on the left side of panel **(B)**], appearing in the crystal, was suggested to be a crystal contact or possibly to be present only under specific circumstances in solution.

ITC was also used to help interpret the X-ray crystallography results for the ANXA2–S100A4 complex (Ecsédi et al., 2017). Previously, the atomic-resolution structure of S100A10 (a highly specific partner of ANXA2) in complex with ANXA2 was published, and a symmetric 2:2 binding mode was reported (Figure 3A) (Oh et al., 2013). Later, we found that, besides S100A10, S100A4 can also bind and regulate the functions of ANXA2 with a different mechanism (Ecsédi et al., 2017). In the latter case, ITC measurements showed an asymmetric 1:2 binding, with one ANXA2 molecule binding the dimeric S100A4. This result was a crucial piece of information after the X-ray structure of this complex was solved and two interacting ANXA2 molecules were identified in the crystal. The binding of one ANXA2 (molecule A) resembled the interaction of ANXA2–S100A10 and involved only one S100A4 monomer, while the second ANXA2 (molecule B) interacted with both major binding pockets of the dimer and even with the intervening cleft (albeit only transiently) (Figure 3B). Since we could rely on ITC results to monitor the stoichiometry in solution, the binding of molecule B was confirmed to be the interaction in solution. The binding of molecule A was suggested to be only a result of crystal-forming contacts, or relevant in certain biological scenarios such as when ANXA2 is associated with phospholipid membranes.

### Analyzing Fuzzy S100 Complexes Using X-Ray Crystallography and Additional *In Vitro* Measurements

**FIGURE 4:**
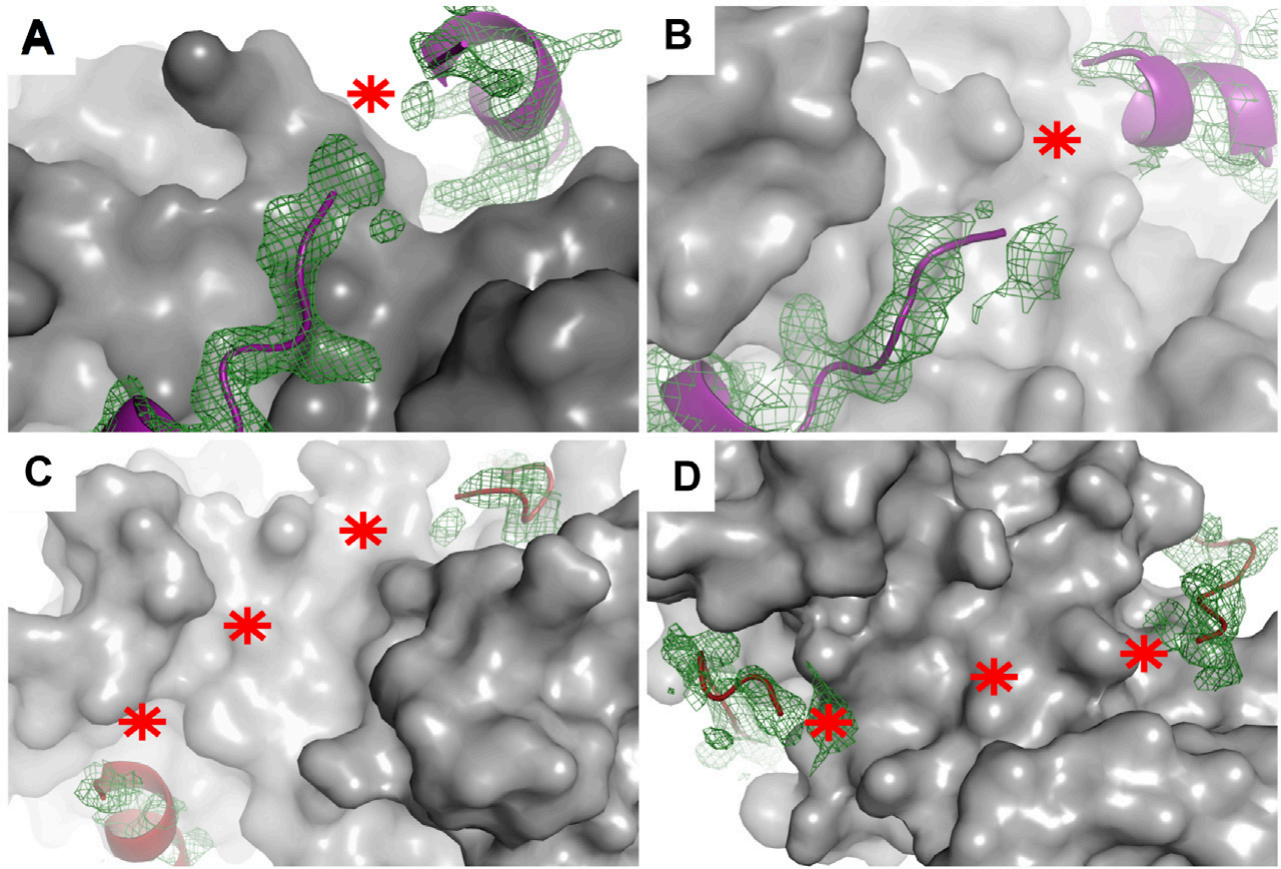
Electron density maps of flexible regions in S100 complexes indicating fuzziness. Structures of **(A)** p53 TAD^17–56^–S100P (PDB: 7NMI), **(B)** p53 TAD^17–56^–S100A4 (PDB: 6T58) (Ecsédi et al., 2020), **(C)** RSK1^683–735^–S100B in one crystal form (PDB: 5CSN), and **(D)** RSK1^683–735^–S100B (PDB: 5CSJ) in another crystal form (Gógl et al., 2016). S100 proteins are displayed as gray surface representations and their partners as deep purple (p53 TAD^17–56^) and red (RSK1^683–735^) ribbons. The green meshes around the partner peptides are simulated annealing Fo–Fc omit maps (contoured at 2*σ*). Red asterisks show the tracks of flexible regions not appearing in the X-ray structures of these complexes.

X-ray crystallography is the method most frequently used to determine the structures of proteins, despite it requires high concentrations of highly pure target proteins from which crystals can be grown. These crystals, like any other crystals, are regular 3D assemblies, but in this case of identical proteins or protein complexes. Depending on the symmetry of this arrangement, all molecules in a crystal lattice show a limited number of unique orientations—and as a result, the diffraction of all individual molecules adds up to yield intensities that are sufficiently strong to be measured, meaning that protein crystals act as amplifiers (Rondeau et al., 2015). Therefore, and specifically because of their lack of uniform conformations, highly mobile targets such as IDPs or fuzzy complexes do not easily organize into crystal lattices, and when found in such lattices, they do not contribute much to the diffraction signal. One strategy here is to remove regions with high conformational freedom, thus increasing the chance of crystal formation as well as strong signal-to-noise data helpful for solving a structure to high resolution. This task is relatively feasible when the N- or C-terminal regions are the disordered regions since truncating them would be expected to have a somewhat low likelihood of perturbing the overall structure (Gógl et al., 2016; Holcomb et al., 2017)—but this task is more difficult when the fuzzy regions are linkers localized between two or more ordered or anchored sections since removing such linkers is relatively likely to change the overall structure of the target, or in the case of interactions, as discussed above, the stoichiometry. Note here that as an alternative, crystallization chaperones can be used as fusion partners to help bypass the difficulties of crystallization. These chaperones can drive crystal and crystal contact formation and at the same time “force” the target molecules into the crystal lattice (Ecsédi et al., 2020; Koide, 2009; Waugh, 2016). Despite successful crystallization of these dynamic targets, either with or without crystallization chaperones, mobile regions in various conformations can still be present in the crystals (Devedjiev, 2015). Since crystallographic electron density maps are generated as an average from a very large number of molecules in the crystal over a long period of time, the highly flexible parts of a protein or a protein complex are associated with poor electron density or are completely missing in the final model (Maveyraud and Mourey, 2020). In this way, highly transient interactions, characteristics of fuzzy complexes, can be recognized since there the electron density tails off and remains visible only at the anchor points. This disappearance of electron density has been found in the cases of several S100 complexes (Figure 4) (Gógl et al., 2016; Ecsédi et al., 2017; Ecsédi et al., 2020). Alternatively, preferred conformations of a heterogeneous ensemble can also be trapped in crystal structures, for example, if they are also establishing packing interactions. In this way, the complex might appear more rigid than when in solution, while other crystals could reveal other states. Therefore, it is always advisable to use various methods to investigate the bound conformations.

In the case of studies where the X-ray structures of the NM2A–S100A4, p53 TAD–S100A4, and RSK1–S100B complexes were determined, the first step in these studies was to find the minimal binding sequences of each partner molecule in order to decrease the flexibility of the complex. The determined minimal binding peptides (NM2A^1894–1937^, p53 TAD^17–56^, RSK1^683–735^) were then used in crystallization trials. Note that, in the case of the complex of S100A4 with p53 TAD, ANXA2 was also included as a crystallization chaperone. These interactions together with those of the ANXA2–S100A4 complex are the currently known examples (with the known structure) of asymmetric binding modes in the S100 protein family, where only one partner molecule binds the dimeric S100. The nature of these interactions is very different. While the NM2A^1894–1934^–S100A4 complex was found to exhibit only minor polymorphism with the whole NM2A peptide visible in the structures (Duelli et al., 2014; Kiss et al., 2012), the other complexes were observed to be more dynamic with sections only transiently or not directly interacting with the S100 partner (dashed lines in Figure 2), representing clamp-like (p53 TAD–S100A4, RSK1–S100B, and ANXA2–S100A4) fuzzy interactions (Gógl et al., 2016; Ecsédi et al., 2017; Ecsédi et al., 2020) (Figure 2).

In these X-ray structures of S100 complexes, the interaction of RSK1 with S100B was found to be the most flexible and diverse. Here, more than one crystal form was observed with slightly different parts of RSK1 binding to S100B. In one structure, the visible parts of RSK1 adopted only coiled conformations, while in others, the peptide also formed helices, but the location of these structural elements varied in the different crystal forms (Figure 2). Circular dichroism (CD) spectroscopy was used to define the structure in solution. Analysis of the CD data, which were processed using the quantitative secondary structure prediction program BeStSel (Micsonai et al., 2015; Micsonai et al., 2018), showed the presence of helices in the complex but also ruled out the possibility of the whole peptide adopting a helical structure upon binding to S100B. Thus, the observed helical elements, appearing in the structures at different locations, cannot exist as helices at the same time (Gógl et al., 2016). CD spectroscopy was also successfully applied in the case of the p53–S100A4 interaction, where the data indicated more helical regions in solution than appearing in the crystal structure (Figure 2), suggesting that the transiently bound sections of p53 are also in helical conformation (Ecsédi et al., 2020).

Besides CD spectroscopy, small-angle X-ray scattering (SAXS) (Bernadó and Svergun, 2012) measurements together with molecular dynamics (MD) simulations (Berendsen et al., 1995; Abraham et al., 2015) can be a useful tool to complement X-ray data in the case of fuzzy complexes (Gógl et al., 2016; Ecsédi et al., 2017; Dudás et al., 2020; Manalastas-Cantos et al., 2021). In SAXS experiments, X-rays are transmitted through the sample and the scattered portion is collected using a 2D detector. The resulting pattern is related to the shape and size of the particles in the sample and gives low-resolution structural information about the proteins in solution (Pelikan et al., 2009; Mertens and Svergun, 2010; Yang et al., 2010; Kikhney and Svergun, 2015). In the case of fuzzy complexes, the ensemble optimization method (EOM) (Bernadó et al., 2007; Mertens and Svergun, 2010; Tria et al., 2015), which is based on the ability to present flexible regions of a protein as ensemble structures, allows one to select the best-fitting solutions from a large computationally generated random set of conformations. EOM and crystallographic data can be easily combined, where flexible regions are modeled and guided by SAXS and MD simulations, while static regions are directly obtained from X-ray crystallography as rigid bodies (Petoukhov et al., 2012). SAXS data can also be directly used for modeling regions not visible in crystal structures (CORAL) (Petoukhov et al., 2012). In the case of the RSK1–S100B interaction, the latter approach was used to obtain more information about this structurally diverse complex (Gógl et al., 2016). Here, the different high-resolution structures obtained from different crystal forms were compared to SAXS data, revealing the predominant conformation in solution. In that structure, only the N- and C-terminal sections of RSK1 could be built in the crystallographic model, and no helix formation is detected. This result together with CD results (which suggested helix formation upon binding) suggested that the flanking region, not visible in the crystal structure, must adopt a more ordered conformation because of steric reasons (Figure 2, marked with a black asterisk).

### Detecting Flexible Regions Using NMR Spectroscopy: Focusing on the p53–S100A4 Complex

Besides X-ray crystallography, cryo-electron microscopy and NMR spectroscopy are capable of determining the structures of macromolecules to high resolution (Kumar et al., 1980; Cavalli et al., 2007). Although the NMR spectroscopy technique has the disadvantage of only being able to be used effectively to analyze macromolecules with molecular weights less than approximately 40 kDa, it does have the advantage of potentially being able to be used to probe the more dynamic aspects of IDP interactions (Schneider et al., 2019) and the great advantage of allowing the structure of a protein and its dynamics to be examined in solution (Purslow et al., 2020). In the case of apo (and Ca^2+^-bound) S100’s and their complexes, NMR spectroscopy has been widely used. NMR spectroscopy has been used to study the binding interfaces (Gógl et al., 2016; Pálfy et al., 2016; Dudás et al., 2020) and the dynamics (Pálfy et al., 2016) of these interactions and to follow the binding-induced changes in the secondary structure (Gógl et al., 2016; Ecsédi et al., 2020). These results gave crucial information and were able to supplement X-ray and other data collected *in vitro*. Moreover, NMR spectroscopy and MD simulation results were also used to determine the structure of the p53 TAD–S100A4 complex to atomic resolution (Dudás et al., 2020), showing the ability to use this method when fuzzy regions must be studied.

One of the most commonly performed protein NMR experiments involves acquiring different ^1^H, ^15^N heteronuclear single quantum coherence (HSQC) (Bodenhausen and Ruben, 1980) spectra of the target. HSQC spectroscopy involves taking 2D NMR measurements of signals appearing only at specific coordinates where there is a correlation of two different but related atomic nuclei, hydrogen atoms and nitrogen atoms in the case of ^1^H, ^15^N HSQC. In proteins, these parameter peaks can be assigned to amino acids exhibiting residue-specific values. Nevertheless, if the chemical environment is disturbed, e.g., during assembly of units into a complex, the amino acid residues involved in the binding would show residue-specific chemical shift perturbations. Due to this effect, the residues involved in the interaction can be detected by performing chemical shift mapping (Williamson, 2013; Williamson and Webb, 2018). Note here that analysis of HSQC spectra can also provide some albeit limited information about the secondary structure of the target molecule, since this technique can be used to discriminate between disordered and structured regions (Dudás et al., 2020). In the case of S100 proteins, several groups have carried out HSQC experiments to determine which of their regions are most crucial for binding. They used different approaches detecting the interacting residues of the targeted S100’s (McClintock and Shaw, 2003; Lee et al., 2008) or of the partners, searching for the minimal binding regions (van Dieck et al., 2009b; Rowell et al., 2012; Gógl et al., 2016; Pálfy et al., 2016). In this way, the flanking N- and C-termini, not involved in the interaction, could be assigned. Furthermore, the ^1^H, ^15^N HSQC spectra of apo and bound p53 TAD and RSK1 peptides showed no changes in chemical shifts in several other residues located in the sequence of the minimal binding peptides (for example, V31, L32, S33 of p53 TAD^17–56^, marked with a dashed line in Figure 2), indicating that these intervening parts remained flexible and did not participate in the interactions.

Since HSQC has low sensitivity, due to peak broadening, minor but conformationally different populations of IDPs and their complexes could remain invisible. To study all states of dynamic systems, Carr–Purcell–Meiboom–Gill (CPMG) relaxation dispersion (Loria et al., 1999; Lundström et al., 2009) and chemical exchange saturation transfer (CEST) (Vallurupalli et al., 2012; Charlier et al., 2017; Schneider et al., 2019) experiments can be carried out. These techniques were also used to determine the kinetics of folding upon complex formation and to determine the structures of intermediate states (Sugase et al., 2007; Schneider et al., 2015).

The secondary structure propensity (SSP) calculation (Marsh et al., 2006) is another easily performed NMR measurement for detecting relaxed regions, since regions involved in binding usually show a high level of structural order, while fuzzy, i.e., transiently interacting, regions often remain disordered. Even measuring the IDRs in the apo form could give some information about their interactions. Thus, we analyzed the SSPs of two different peptides, capable of interacting with S100A4, namely, the p53 TAD^1–60^ (Dudás et al., 2020) and NM2A^1893–1937^ (Pálfy et al., 2016) fragments. SSP data showed three small regions with high helical propensity in the case of p53 TAD (Dudás et al., 2020; Ecsédi et al., 2020), while a more extended sequence of the NM2A peptide was shown to form a transient α-helix (Pálfy et al., 2016). Based on these data, one could assume that regions with higher helix propensities represent the binding surfaces of these peptides, while lower ordered sections remain flexible in the complexes. This correlation was evident in the case of p53 TAD, but only partially in the case of NM2A when the high-resolution X-ray structures of their complexes with S100A4 were also analyzed. The transient helices, defined in the SSP experiments, were found to nicely correlate with the stable helices appearing in the X-ray structures (Elliott et al., 2012; Kiss et al., 2012; Duelli et al., 2014; Ecsédi et al., 2020) (Figure 2), and it was agreed that they contain the main binding surfaces of the two peptides. The SSP-predicted disordered regions, on the contrary, were found to function differently in these cases. The linkers between the three p53 TAD helices remained flexible and only transiently interacted with S100A4 causing a clamping fuzzy interaction, while the C-terminal section of NM2A, also predicted to be disordered in the apo form, bound to S100A4 with high specificity, forming a polymorphic complex with S100A4 (see Figure 1 for more examples).

**FIGURE 5:**
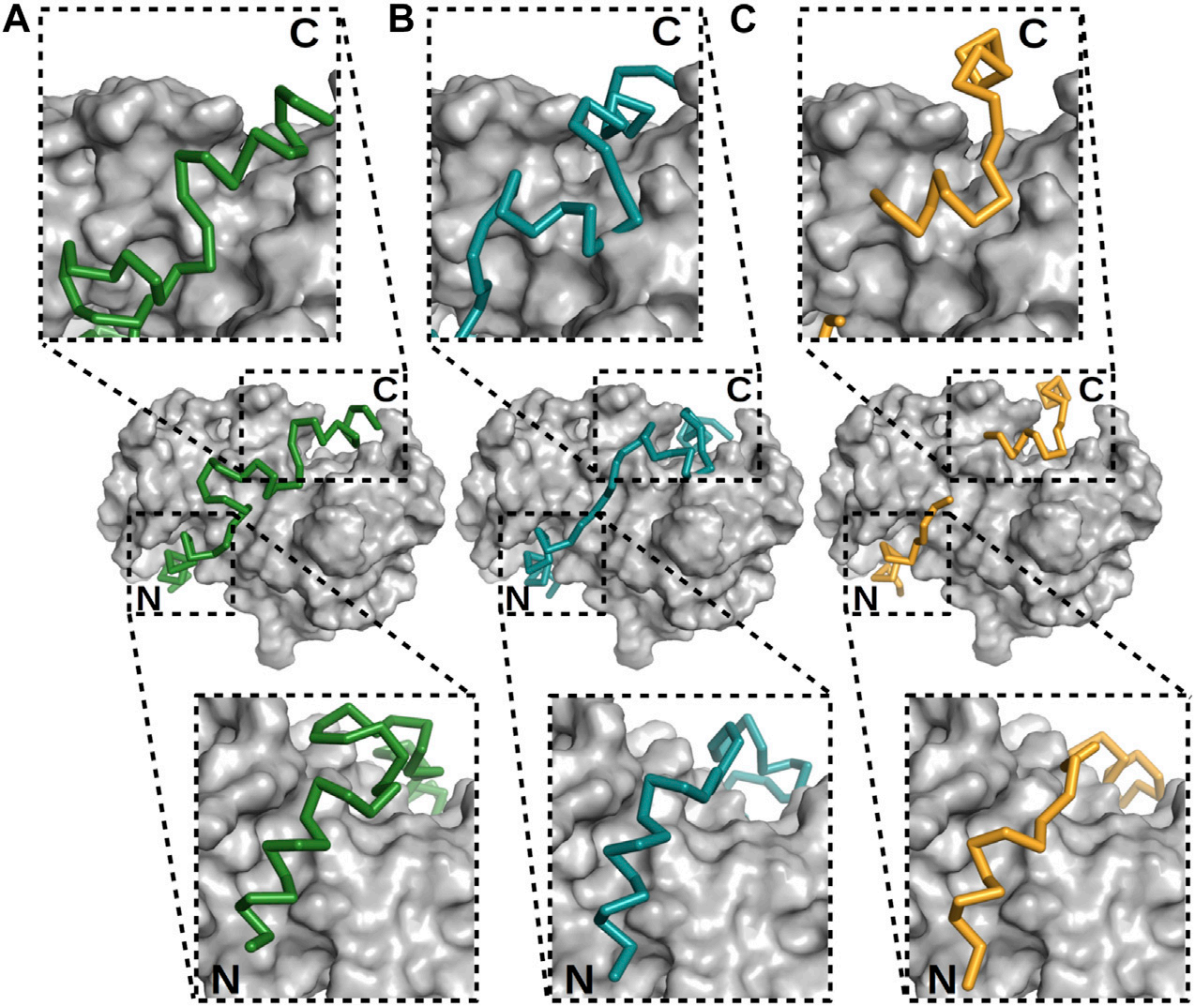
Different modes of binding of p53 TAD to S100A4. **(A, B)** The two best models built, using NMR data (Dudás et al., 2020), and **(C)** X-ray crystal structure of the p53 TAD^17–56^–S100A4 complex (Ecsédi et al., 2020). p53 is shown as alpha-carbon traces in different colors, while S100A4 is shown as surface representations in gray. The labels N and C mark the locations of the N- and C-termini of the p53 peptide. The binding of the N-terminus is nearly identical in these three cases, but the C-terminus and the linker connecting the ordered regions differ, showing the different binding modes of p53. In the crystal structure determination, parts of the linker were not visible, indicative of its flexibility.

A powerful NMR tool available for studying the structural ensemble of interactions is based on nuclear Overhauser enhancement (NOE) constraints, which are crucial for solving structures when using NMR. However, these constraints can be used in the cases of IDPs and fuzzy regions only with limitations, since they were established for well-folded proteins, particularly those featuring long-lived secondary structures (Kozak and Kurzbach, 2021). In the case of more transient systems such as fuzzy complexes, other techniques must be considered; these techniques include paramagnetic relaxation enhancement (PRE), which allows for the estimation of long-range distances (up to 35 Å) (Solomon, 1955; Olivieri et al., 2018), or a complex MD-NMR approach, where MD simulations can complement spectroscopy data only when few experimental constraints are available (Stocker and van Gunsteren, 2000; Robustelli et al., 2010; Dudás et al., 2020). A similar approach was used to help determine the structure of the p53 TAD^1–60^–S100A4 complex (Dudás et al., 2020). The X-ray and NMR structures of p53 TAD^17–56^–S100A4 and p53 TAD^1–60^–S100A4 complexes were analyzed together leading to a detailed description of this interaction (Figure 5). These results also indicated the formation of three helices upon binding; but note that analysis of only the crystal structure would not clearly indicate the specific helix primarily responsible for binding. NMR data showed lower flexibility for L22–P27 (N-terminal helix) and S46–I50 (third helix) than for their surroundings, indicating these regions to be the primary interaction sites.

### Function and Fuzziness in the S100 Family

The sites on the partner proteins that bind S100’s are often also used to bind other proteins. For example, the NM2A binding site is involved in oligomerization (filament formation) (Kiss et al., 2012; Ecsédi et al., 2018); the interacting region of RSK1 might be bound to ERK2, as well as being part of an autoinhibitory segment (Gógl et al., 2016); the p53 binding fragment is usually occupied with other regulatory partners such as MDM2 (Raj and Attardi, 2017); the binding region in ezrin is involved in autoinhibition (Biri-Kovács et al., 2017); and the binding site on the ANXA2 protein is partially in a self-bound state in the free form, helping to stabilize the protein (Ecsédi et al., 2017). Non-catalytic S100 proteins can alter the functions of their partners by interfering with these or similar alternative interactions. Moreover, most S100 proteins have been indicated to interact with multiple partners, and most of these partners have shown detectable interactions with more than one S100 protein (Simon et al., 2020). This promiscuity might allow different members of the S100 protein family to substitute the functions of each other in certain circumstances, for example, when the concentration of a particular S100 member is low, which is usually the case since S100 proteins are expressed in tissue- and cell-specific manners (Donato et al., 2013). To be able to bind a wide variety of ligands without optimal binding motifs, S100 proteins may have developed relatively permissive binding sites leading to more flexible interactions (Kaneko et al., 2012). Since structural heterogeneity in protein complexes may weaken the sequence constraints for a specific partnership (Fuxreiter et al., 2012; Fuxreiter, 2018), the appearance of fuzziness in S100 complexes might be the consequence of the S100 family members maintaining their ability to bind multiple partners. Note here that fuzziness can also be beneficial for achieving relatively low–specificity interactions, since a disordered region connecting two bound ordered domains contributes to the binding by reducing the entropic penalty (Tompa and Fuxreiter, 2008).

In the case of flexible complexes, different conformational states of such complexes might also be responsible for different functions. This feature was suggested when the RSK1–S100B interaction was studied (Gógl et al., 2016). In that study, one of the observed states was found to be relatively static, and based on an *in vitro* analysis, it was suggested to possibly represent an autoinhibitory RSK1 complex. The other, more fuzzy complex was suggested to be a transitional state, with RSK1 in a bound form but without its activity being affected.

### Conclusion and the Importance of Solving the Fuzzy Structures of Multiple S100 Complexes

Undoubtedly, there are fuzzy protein complexes, but studying their structures is a challenge even today. Here, we have discussed the suitability of using NMR spectroscopy and X-ray crystallography together with various other techniques (CD, ITC, SAXS, and MD simulations) for studying these transient interactions. As we experienced with S100 complexes, these methods can and should be used complementarily to obtain as detailed information about flexible complexes as possible. Such use of multiple techniques is important because in numerous cases (like in the S100 family), fuzziness indicates the existence of multiple binding partners, and by analyzing each interaction thoroughly, partner-specific surfaces could be recognized. Obtaining as much detailed knowledge of these surfaces as possible could aid the development of inhibitor variants, which could be used to selectively limit the binding of the targeted protein, leaving other functions intact. In this way, more effective therapeutics can be developed. In this respect, the studies of S100A4 complexes could be giving a glimpse into how new inhibitors might be developed and work in the future. S100A4 has been shown to interact with NM2A (Ecsédi et al., 2018; Kiss et al., 2012; Elliott et al., 2012), p53 (Dudás et al., 2020; van Dieck et al., 2009b; Orre et al., 2013), and ANXA2 (Ecsédi et al., 2017; Semov et al., 2005) with different affinities, namely, relatively high affinity for NM2A (K_d_ < 1 nM) and low affinity for p53 and ANXA2 (K_d_ ∼ 2 µM). By interacting with these proteins, S100A4 can regulate or even disrupt their normal function. The crystal structures of these complexes have shown that each partner occupies the binding pockets of the S100A4 dimer but that they use the intervening cleft differently: p53 and ANXA2 remain highly flexible outside of the canonical binding sites, while the NM2A–S100A4 complex is more compact, anchoring NM2A also along the intervening cleft (Figure 2). These additional contacts may also account for, as discussed, the greater affinity of NM2A than of p53 and ANXA2 for S100A4. This structural difference could be utilized, since a molecule competing with the sequence of NM2A contacting the surface outside of the S100A4 binding pockets might inhibit or at least disrupt the formation of the NM2A–S100A4 complex while at the same time allowing p53 and ANXA2 to interact with S100A4 in an uninterrupted manner (Bresnick, 2018; Hua et al., 2020). By understanding the structures of more S100 complexes, partner-specific binding surfaces similar to those of S100A4 could be located. Future structural biology studies may also shed more light on the surprising diversity and still unrecognized functions of the evolutionary young S100 family.
